# A notodontid novelty: *Theroa zethus* caterpillars use behavior and anti-predator weaponry to disarm host plants

**DOI:** 10.1371/journal.pone.0218994

**Published:** 2019-07-10

**Authors:** David E. Dussourd, Madalyn Van Valkenburg, Kalavathy Rajan, David L. Wagner

**Affiliations:** 1 Department of Biology, University of Central Arkansas, Conway, Arkansas, United States of America; 2 Department of Biological and Agricultural Engineering, University of Arkansas, Fayetteville, Arkansas, United States of America; 3 Department of Ecology & Evolutionary Biology, University of Connecticut, Storrs, Connecticut, United States of America; Universidade de Sao Paulo, BRAZIL

## Abstract

Unlike most notodontids, *Theroa zethus* larvae feed on plants that emit copious latex when damaged. To determine how the larvae overcome this defense, we filmed final instars on poinsettia, *Euphorbia pulcherrima*, then simulated their behaviors and tested how the behaviors individually and combined affect latex exudation. Larvae initially scraped the stem, petiole, or midrib with their mandibles, then secreted acid from their ventral eversible gland (VEG) onto the abraded surface. Scraping facilitated acid penetration by disrupting the waxy cuticle. As the acid softened tissues, the larvae used their mandibles to compress the plant repeatedly, thereby rupturing the latex canals. Scraping, acid application, and compression created withered furrows that greatly diminished latex exudation distal to the furrows where the larvae invariably fed. The VEG in notodontids ordinarily serves to deter predators; when attacked, larvae spray acid aimed directly at the assailant. Using HPLC, we documented that the VEG secretion of *T*. *zethus* contains 30% formic acid (6.53M) with small amounts of butyric acid (0.05M). When applied to poinsettia petioles, the acids caused a similar reduction in latex outflow as VEG secretion milked from larvae. VEG acid could disrupt latex canals in part by stimulating the normal acid-growth mechanism employed by plants to loosen walls for cell elongation. Histological examination of cross sections in poinsettia midribs confirmed that cell walls within furrows were often highly distorted as expected if VEG acids weaken walls. *Theroa zethus* is the only notodontid caterpillar known to use mandibular scraping and VEG acid to disable plant defenses. However, we document that mandibular constriction of petioles occurs also in other notodontids including species that feed on hardwood trees. This capability may represent a pre-adaptation that facilitated the host shift in the *Theroa* lineage onto latex-bearing plants by enabling larvae to deactivate laticifers with minimal latex contact.

## Introduction

Most species of herbivorous insects specialize on plants within a single family, often utilizing only one or a few species [1]. Closely related insects tend to consume taxonomically-related plants [2, 3]. However, exceptions to this pattern are numerous. How herbivores tolerate or disarm the defenses of unusual hosts is poorly understood. Biochemical and behavioral adaptations of herbivores to typical food plants, such as danaines on milkweeds or pierids on crucifers, have been studied extensively in many cases [4–6], but herbivore adaptations to distantly related unusual hosts have received less attention.

Caterpillars within the Notodontidae feed primarily on leaves of woody trees and shrubs; temperate lineages are particularly diverse on Fagaceae and Salicaceae, but some utilize hosts that differ markedly in defenses and ecology [7]. A notable example, *Theroa zethus* feeds on ephemeral herbs in the Euphorbiaceae (euphorbs) that germinate and grow rapidly in response to seasonal monsoon rains in the American Southwest. When damaged, their *Euphorbia* and *Chamaesyce* host plants emit copious quantities of latex [8], a defensive fluid known to be poisonous in diverse plants [9–12], including in related euphorb species [13–15]. The latex coagulates as it exudes from the plant [16], potentially entrapping or gumming up insect herbivores [10, 17, 18]. *Theroa zethus* is the only known member of its genus and one of just a few notodontids in North America that feed on hosts protected by latex canals (laticifers) or other secretory canals [19–21], although notodontids that feed on euphorbs or other laticiferous plants have been reported elsewhere, mostly in the Old World [22].

Latex in euphorbs is stored under pressure within living cells that form elongate branching tubes [23–27]. In mature plants, the laticifers occur in the stem, petiole, and midrib and tend to follow the lateral and minor veins in the leaf [25, 27, 28]. Feeding folivores rupture the canals, which causes the immediate flow of latex from high pressure in the canals to low pressure at the breach [29, 30]. *Theroa zethus* larvae disarm the laticifers by co-opting their defensive weaponry: they secrete concentrated acid onto the plant surface from a gland located ventrally between the head and prothoracic legs [8]. This gland is variously called the ventral eversible gland [31–33], prothoracic gland [34, 35], adenosma [36], cervical gland [20, 37] or repugnatorial gland [38]; we will use the term ventral eversible gland (VEG). In most notodontids, the VEG serves to deter predators; when disturbed, larvae spray acid precisely aimed at the attacker [8, 34, 35, 38].

**Fig 1:**
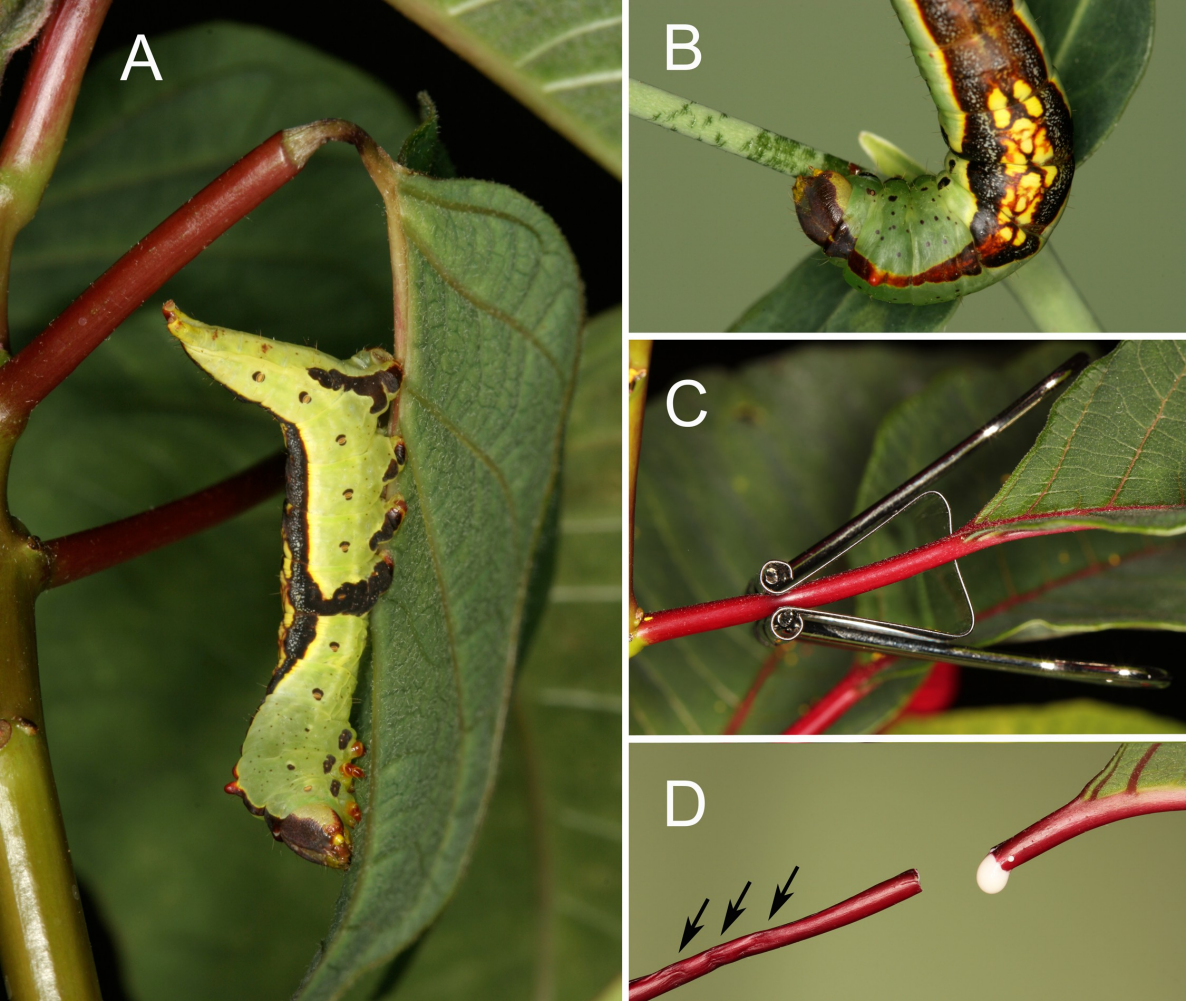
Natural and artificial girdles in euphorb petioles. **(A)** Finished girdle in a poinsettia petiole created by a final instar *Theroa zethus*. (B) *Theroa zethus* larva scraping the surface of a *Euphorbia corollata* stem. Scratch marks created by mandibular teeth are readily visible as dark green parallel lines. (C) Binder clip used to compress poinsettia petioles. (D) Effect of VEG acid and petiole compression on latex outflow from the petiole stub (left) and from the leaf blade (right). Arrows indicate locations where the petiole was compressed horizontally and vertically three times by the binder clip. Only outflow from the petiole stub was reduced significantly.

*Theroa zethus* application of VEG acid to the plant surface causes visible withering, producing conspicuous furrows in leaf midribs or girdles that encircle stems and petioles (Fig 1A). Both furrows and girdles reduce distal latex emission [8]. Previous work has documented that *T*. *zethus* larvae with blocked VEG glands are unable to create furrows or girdles. As a result, their growth is substantially reduced on poinsettia leaves with intact laticifers, but not on excised leaves with depressurized laticifers [8]. VEG secretion milked from *T*. *zethus* larvae and applied to the midrib surface creates withered furrows and decreases distal exudation [8]. However, the VEG secretion by itself requires a lengthy period to be effective (24 hours in Dussourd [8]). The larvae also mandibulate and compress the plant surface. Do these behaviors enhance the efficacy of the secreted acids?

This study has four principal objectives:

1. To document behaviors employed by *T*. *zethus* larvae while producing furrows and girdles and to test how the behaviors individually and together affect latex exudation.
2. To identify and quantify acids present in *T*. *zethus* VEG secretion and to test if the acids by themselves disrupt poinsettia laticifers as effectively as the VEG secretion.
3. To test a potential mechanism for how VEG acid disrupts laticifers. Specifically, we test if cells within *T*. *zethus* furrows have deformed walls as predicted if VEG acid activates the plant’s own acid growth mechanism for loosening walls.
4. To test if the behaviors employed by *T*. *zethus* are utilized also by other notodontid species.

## Materials and methods

### Study organisms

*Theroa zethus* larvae were imported from southern Arizona to Arkansas under USDA APHIS permits P526P-12-00188 and P526P-14-03555. Because only caterpillars and plants were used in this study, no approval was required from Institutional Animal Care and Use Committees. In southeastern Arizona, *T*. *zethus* larvae feed on *Euphorbia cyathophora*, *E*. *dentata*, *Chamaesyce hyssopifolia*, and *C*. *serpyllifolia* [8]; in the lab, the larvae readily consume other *Euphorbia* and *Chamaesyce* as well. Poinsettia, *Euphorbia pulcherrima*, was chosen for experimental work because the leaves have robust midribs and petioles that release copious latex; *T*. *zethus* larvae must employ VEG acid to effectively disable the laticifer system [8]. The behavior of final instar larvae on poinsettia resembles behavior on natural hosts (S1–S4 Movies). Potted mature poinsettia grown from cuttings in a greenhouse were used in all experiments (Eckespoint Classic Red for histology, Poinsettia C Classic Red for all other experiments). In each experiment, plants were randomly assigned to a treatment group and only a single petiole (~3.5 mm wide) of a mature intact green leaf was used per plant.

### Effect of abrasion on acid penetration

Before feeding on a poinsettia leaf, *T*. *zethus* larvae mandibulate the petiole or midrib extensively, alternating between scraping the epidermis with their mandibles and applying acid with their VEG opening pressed against the plant surface (S1 Movie). To test if surface abrasion facilitates acid penetration resulting in a greater reduction in distal latex outflow, we lightly rubbed ~1 cm of the dorsal surface of poinsettia petioles ten times with fine sandpaper (3000 grit) halfway between the stem and leaf blade. A drop of water was used to lubricate the sandpaper to mimic fluid (presumably saliva) secreted by *T*. *zethus* larvae [8]. The petioles have a relatively flat dorsal surface; abrasion and subsequent drying with a paper towel usually did not elicit latex release. Plant physiologists have similarly abraded the cuticle of hypocotyls with emery cloth to enhance acid penetration [39]. Four treatments were tested: 5 μL VEG secretion or 5 μL water (pH 7.0) applied to abraded petioles, and 5 μL VEG secretion or water applied to control petioles not abraded (n = 10 plants/treatment). VEG secretion from final instar *T*. *zethus* larvae was obtained by inserting their head into glass vials and pinching their abdomen with forceps to trigger VEG spraying. Approximately 2.5 hours after petioles were treated with VEG secretion or water, they were severed ~2 mm from the leaf blade and all latex flowing from the petiole stub was collected onto filter paper and weighed. The goal was to test if the solutions applied to the petiole surface disrupt laticifers within the petiole preventing latex flow into the leaf.

### Effect of petiole compression on latex exudation

As repeated application of VEG acid softens the petiole [8], *T*. *zethus* larvae increasingly use their mandibles not to scrape, but to grasp and compress the petiole (S3 Movie). We tested if compression blocks latex flow through laticifers in poinsettia petioles by clamping the petioles in the middle repeatedly with a used medium binder clip (Fig 1C). The clip was placed on the petiole, then immediately released; it visibly compressed the petiole surface, but usually did not elicit latex release, similar to the compressions of *T*. *zethus* mandibles. Petioles were compressed horizontally and vertically three times, each compression 2–3 mm distal to the previous one. Maximum force generated by the binder clip (8.245 ± 0.015 N) was measured with a custom-made force sensor composed of a 5 kg load cell connected to an amplifier unit and a multimeter. Although tiny caterpillar mandibles generate a much smaller adductor force (~0.4 N for fifth instar *Heliothis virescens* [40]), the clip produces a standard force useful for measuring the impact of compression on laticifers in petioles with and without VEG acid.

Four treatments were tested: 1) 5μL VEG secretion applied to abraded petioles that were compressed one hour later at the application site, 2) 5μL VEG secretion left on abraded petioles for one hour without compression, 3) petioles compressed without abrasion or acid treatment, and 4) an unaltered control with no acid or compression (n = 10 plants/treatment). Immediately after compression for treatments 1 and 3, or one hour after VEG secretion application for treatment 2, petioles were severed near the base of the leaf blade and latex flowing out from the petiole was collected onto filter paper and weighed. To determine how latex pressures within the leaf blade were affected, latex flowing from the severed leaf was also collected separately and weighed.

### Effect of repeated compression of petioles on latex exudation

*T*. *zethus* caterpillars ordinarily compress poinsettia petioles dozens of times. The larva filmed in S3 Movie, for example, constricted the petiole with over 175 distinct contractions of its mandibles, only a few of which are shown in the short video clip. On euphorbs with narrow stems, larvae often constrict the stem with a sequence of compressions produced in a row as the larva moves towards the apex [8]. To test if compressing the petiole repeatedly reduces latex emission more effectively than fewer compressions, petioles were clamped 20 times (instead of six previously) using the same binder clip as before. Horizontal and vertical compressions were alternated. Compressions were made in the petiole from the stem towards the leaf or from the leaf towards the stem; each successive compression was made 1–2 mm from the previous one. Immediately after the twentieth compression, the petiole was severed near the leaf base and latex exuding from the petiole stub and from the leaf were weighed. Unaltered plants served as a control (n = 10 plants/treatment). This experiment tests not only if multiple compressions reduce latex pressures more effectively than fewer compressions, but also if the direction of compressions influences how effectively they block latex flow into the leaf and drain latex from the leaf.

### Chemistry of VEG secretion

Previous studies of notodontid VEG secretions have documented the presence of formic acid sometimes supplemented with smaller amounts of acetic acid [34, 35, 37, 41, 42]. We tested for the presence of formic, acetic, propionic, butyric and isobutyric acid using HPLC. VEG secretion was collected from three sets of 20 final instar *T*. *zethus* larvae. Samples were diluted 4X with de-ionized water, filtered through a 0.22 μm membrane, then three 10 μL aliquots of each sample were analyzed with a Waters 2695 separations module equipped with an Aminex HPX-87H ion exchange column (7.8 mm × 30 mm) heated to 55°C (Bio-Rad Laboratories, Inc. Hercules, CA). The mobile phase consisted of 0.005M H_2_SO_4_ eluting at a flow rate of 0.6mL/min. Formic and butyric acids were detected using a Waters 2996 photodiode array detector set at 210 nm (Waters, Milford, MA). The identity of acids was confirmed by comparing retention times with standards of formic acid and butyric acid (Sigma Aldrich, St. Louis, Missouri, USA) and by spiking VEG samples with these acids. Acid concentrations were quantified by using in-house calibration curves.

To test if a reconstituted acid solution that matches *T*. *zethus* VEG secretion (6.53M formic acid and 0.05M butyric acid) disrupts poinsettia laticifers as effectively as the VEG secretion, we applied 5 μL of *T*. *zethus* VEG secretion, reconstituted acids, or pH 7.0 water to the middle of poinsettia petioles abraded on the dorsal surface with fine sandpaper (3000 grit) as described previously. After ~2.5 hours, the petioles were severed at the base of the leaf and latex exuding from the petiole stubs was collected onto filter paper and weighed (n = 10 replicates/treatment).

Finally, to determine if formic or butyric acid reduced latex outflow, we placed 5 μL of 6.53M formic acid + 0.05M butyric acid, 6.53M formic acid alone, 0.05M butyric acid alone, or pH 7.0 water on abraded poinsettia petioles and again measured latex outflow after 2.5 hours from severed petioles (n = 10 replicates/treatment).

The pH of VEG samples and reconstituted acid samples were measured with a Sartorius pH Core meter with a Van London micro pH electrode #5473901. Three 100 μL samples/solution were each tested three times.

### Histology of *T*. *zethus* furrows

To determine how VEG secretions and saliva of final instar *T*. *zethus* affect the morphology of poinsettia tissues, cross-sections through midrib furrows were prepared using standard histological techniques (based on Ganong et al. [43] and described in detail in Van Valkenburg [44]). *Theroa zethus* larvae appear to secrete saliva as well as VEG fluid onto midrib furrows [8], perhaps to provide lubrication for scraping the plant surface. We compared midrib furrows created by intact larvae, by larvae with their VEG orifice blocked, and by larvae with their spinneret cauterized to eliminate saliva secretion from the labial salivary glands. As described previously [8], the VEG was blocked with Permatex superglue containing ethyl cyanoacrylate and the spinnerets were cauterized using a Bonart ART-E1 electrosurgery unit with a sharpened fine tip. Larvae with their VEG opening blocked still pressed the VEG opening against the plant surface, but a furrow was not produced [8].

After larvae initiated feeding, midrib furrows were excised and fixed overnight (>16 hrs) in HistoChoice tissue fixative (Amresco, Solon, Ohio, USA) under vacuum. The samples were then dehydrated in an ethanol series and cleared using HistoChoice clearing agent (BioExpress, Kaysville, UT). Next, midribs were infiltrated with paraffin wax (Paraplast plus, Fisher Scientific, Pittsburgh, PA), sectioned, and stained with toluidine blue. The resulting slides were photographed with an Olympus BX40 microscope. Cross-sections inside and outside midrib furrows for each of the three treatments were compared by counting the number of convex cells (all interior angles of the cell wall are less than or equal to 180°) and concave cells (an interior angle measures more than 180°) intercepted by a transect from the epidermis to the phloem (n = 3 furrows/treatment).

### Related behaviors in other notodontid species

To determine if mandibular scraping, VEG acid application, and compression are employed by other species, we photographed and filmed final instar larvae of the following six notodontid species: *Praeschausia zapata* and *Lochmaeus manteo*, which are classified with *Theroa* in the Heterocampinae; *Datana perspicua*, *Nadata gibbosa*, and *Peridea angulosa* in the Phalerinae, and *Paraeschra* (= *Hyperaeschra) georgica* in the Notodontinae [20]. *Praeschausia zapata* larvae can be found in southern Arizona feeding on the same individual *Chamaesyce hyssopifolia* plants as *T*. *zethus* (Dussourd unpub. obs.). We photographed *P*. *zapata* in the field on *C*. *hyssopifolia* and filmed larvae in the lab on mature potted *C*. *nutans*. *Datana perspicua* larvae feed on sumac (*Rhus)* and other Anacardiaceae [21] that emit exudates from intercellular ducts when damaged [45, 46]. We photographed a cluster of this social species placed on smooth sumac, *Rhus glabra*. The final four species, *Lochmaeus manteo*, *Nadata gibbosa*, *Peridea angulosa* and *Paraeschra georgica* all feed on hardwoods with oak (*Quercus*, Fagaceae) being the preferred or sole host [21]. Oak leaves do not emit visible exudate when damaged and lack laticifers and ducts [47, 48]. We photographed and filmed *N*. *gibbosa* on detached branches of post oak (*Q*. *stellata*) and southern red oak (*Q*. *falcata*) with the cut end in water and the other three species on detached branches of water oak (*Quercus nigra*).

### Statistical analyses

The wet weight of latex emitted by poinsettia leaves was measured in the two experiments that analyzed the effects of compression on latex outflow. In each experiment, treatments were compared with a one-way ANOVA followed by pairwise Tukey tests using JMP v. 11 (SAS Institute Inc., Cary, NC). In each of the five experiments that measured latex emitted by petioles, variances were unequal so Welch’s ANOVAs were used instead with Games-Howell tests selected for paired *post hoc* comparisons [49]. The data were square-root transformed to satisfy the normality assumption with the fourth experiment comparing VEG acid, a reconstituted acid solution, and the water control. Finally, for the histology data, paired *t*-tests were used to compare the proportion of concave cells within a furrow vs. outside a furrow. All graphs present means ± 1 standard error with α < 0.05 employed for statistical significance.

### Photographing and filming caterpillar behavior

*Theroa zethus* and *Praeschausia zapata* behaviors were recorded in the lab on potted euphorbs with intact latex canals using a Wild M400 photomacroscope outfitted with a Canon T3i or T4i camera. Photographs of *Datana perspicua* and *P*. *zapata* in the field were taken using the same cameras with a Canon 100 mm macro lens. Other notodontid species were photographed and filmed in the lab on detached oak branches; the Canon cameras, 100 mm macro lens or Canon MP-E 65 mm lens, and various light sources including ring lights and LED light panels were used.

## Results

### Effect of abrasion on acid penetration

**Fig 2:**
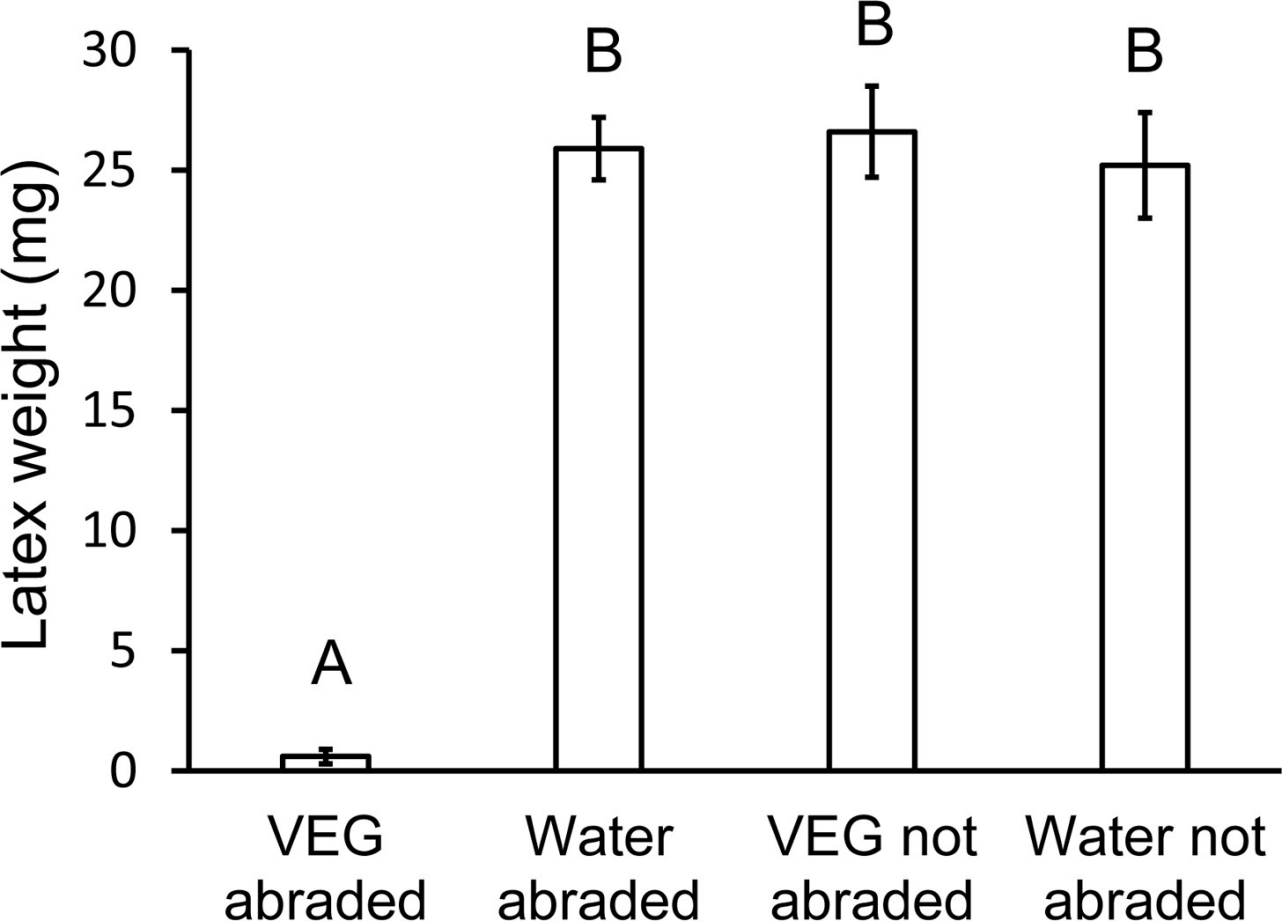
Effect of abrasion on latex exudation from poinsettia petioles. Half of the petioles were rubbed on the dorsal surface with sandpaper. The petioles then received 5μL of either VEG secretion or water. After 2.5 hours, the petioles were severed and the wet weight of latex exuding from each petiole stub was weighed. Abrasion facilitated VEG acid penetration resulting in nearly complete elimination of latex outflow from petioles. Bars with different letters differ significantly at *P* < 0.05 using Games-Howell tests.

Petioles in the four treatments differed significantly in the weight of latex emitted (Fig 2, Welch’s ANOVA, F_3,15.8_ = 201, *P* < 0.0001). Abrading the petiole surface before applying VEG acid resulted in rapid withering and reduced outflow of latex from the petiole. Even though the VEG secretion was applied only to the dorsal surface of the petiole, it blocked latex flow from the stem to the leaf in laticifers throughout the petiole. The reduced outflow could not be attributed to abrasion alone because abraded petioles treated with water emitted comparable quantities of latex as the non-abraded controls (Fig 2). VEG secretion by itself likewise was ineffective because petioles were severed after just 2.5 hours; if the acid was allowed to penetrate for a longer period (such as 24 hours [8]), a reduction in latex outflow would have occurred.

### Effect of petiole compression on latex exudation

**Fig 3:**
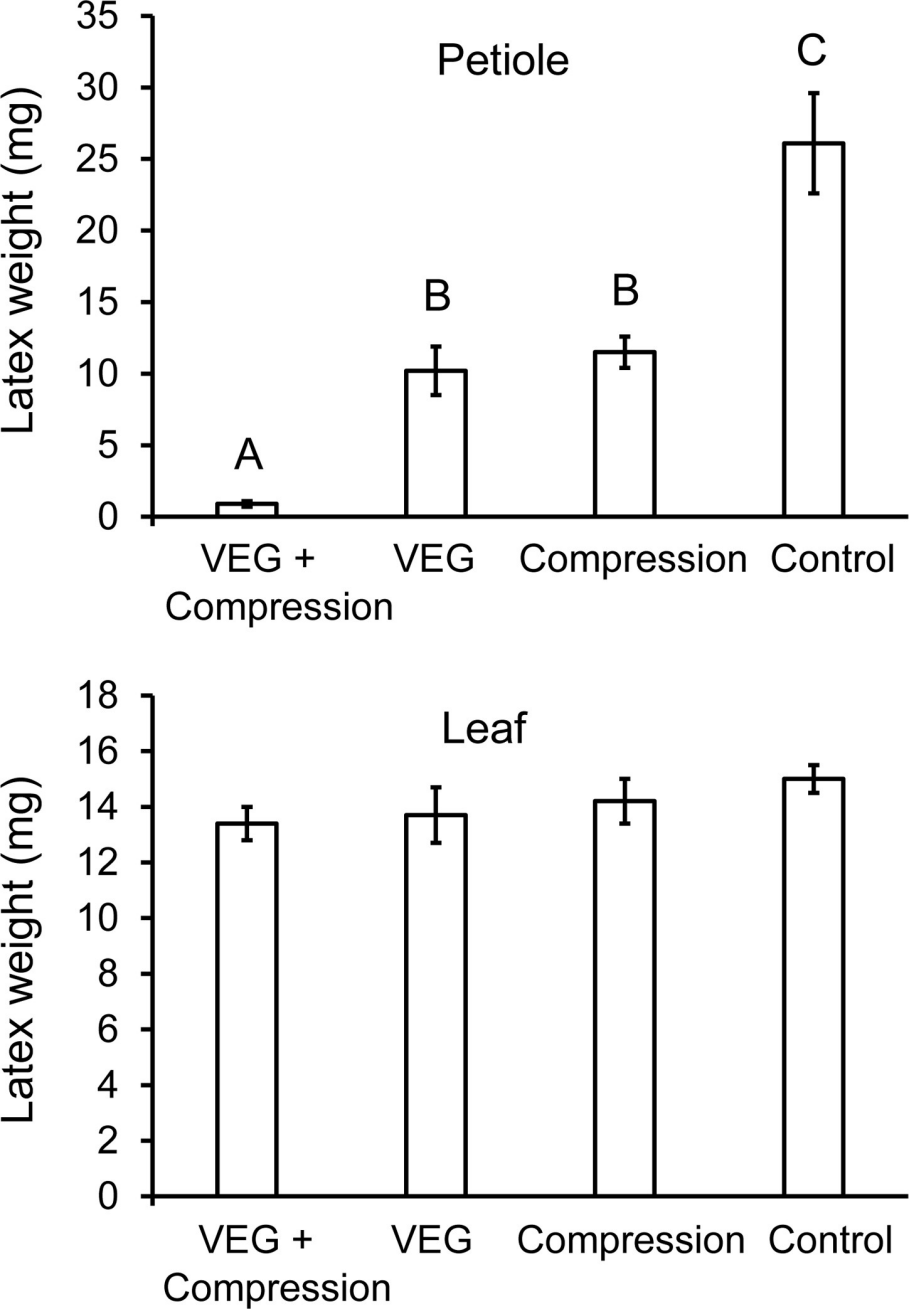
Effect of compression on latex exudation from poinsettia petioles and leaves. The petioles were treated either with VEG acid for an hour (following surface abrasion), compression with a binder clip, or both acid and compression. Acid treatment followed by compression reduced the wet weight of latex emitted by the petioles more effectively than either acid or compression tested alone (top). In contrast, neither acid nor compressions significantly reduced latex outflow from the leaf blades (bottom). Bars with different letters differ significantly at *P* < 0.05 using Games-Howell tests.

Weights of latex emitted by poinsettia petioles differed significantly in the four treatments (Fig 3, Welch’s ANOVA, F_3,15.5_ = 53.4, *P* < 0.0001). Both VEG acid and compression independently reduced latex emission relative to the control, decreasing levels by over 50%. When combined, VEG acid and compression eliminated almost all latex outflow from the petiole (Figs 1D and 3). Leaf latex levels, in contrast, did not differ significantly (ANOVA, F_3,36_ = 0.89, *P* > 0.05). Neither VEG acid nor compression substantially altered latex outflow from the leaf. Thus, acid and compressions effectively blocked latex flow from the stem to the leaf, but caused minimal drainage of latex from the leaf.

### Effect of repeated compression of petioles on latex exudation

**Fig 4:**
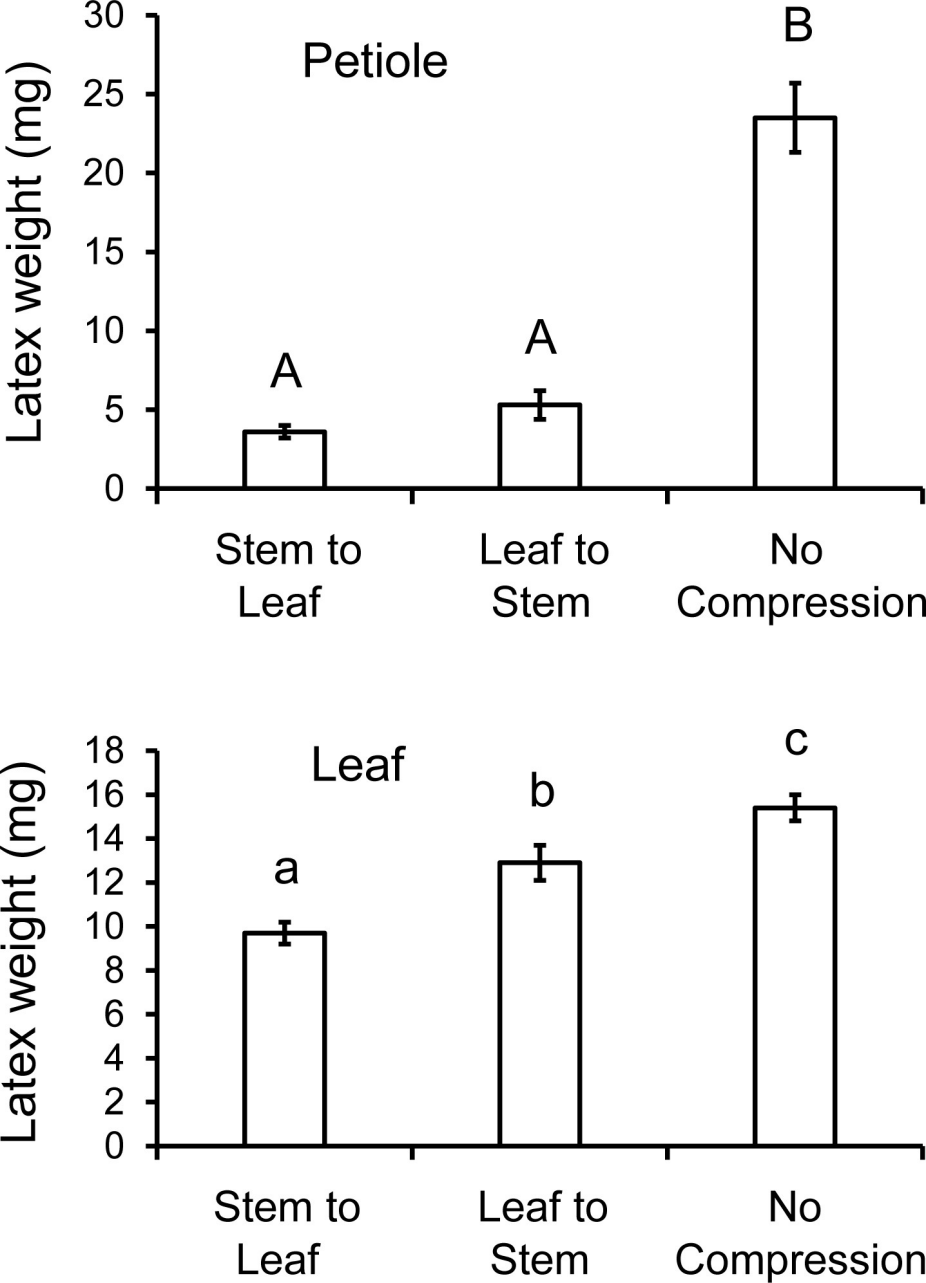
Effect of multiple compressions on latex outflow from poinsettia petioles and leaves. Petioles were compressed 20 times with a binder clip, then they were severed and the wet weight of latex exuding from the petiole stub (top) and leaf blade (bottom) was measured. Compressions made in the petiole from stem to leaf were especially effective at draining latex from the leaf blade. Bars with different letters differ significantly at *P* < 0.05 using Games-Howell pairwise tests (top) or Tukey tests (bottom).

Compression significantly reduced latex emission from both petioles (Fig 4, Welch’s ANOVA, F_2,14.2_ = 39.5, *P* < 0.0001) and leaves (ANOVA, F_2,27_ = 19.4, *P* < 0.0001). Latex outflow from the petiole was decreased similarly relative to the control whether the compressions were made from the stem towards the leaf or in the opposite direction (*P* < 0.05, Games-Howell tests). In both cases, the compressions prevented latex flow through the petiole. Twenty compressions in either direction caused a greater reduction in latex outflow from petioles than six compressions (without abrasion and VEG acid) in the previous experiment (*P* < 0.001, *t*-tests). In contrast, with leaf latex, compressions made from the stem towards the leaf more effectively reduced latex outflow from the leaf than compressions from leaf to stem (*P* < 0.05, Tukey test). The stem-to-leaf compressions repeatedly damaged laticifers extending into the leaf, thus progressively draining latex from the leaf. With compressions made from leaf to stem, the initial compressions close to the leaf blade isolated the laticifers in the leaf from additional compressions made in the petiole closer to the stem. Since the compressions caused minimal external exudation of latex (0.8 ± 0.4 mg latex for the two compression treatments combined), the reduction in leaf latex relative to the control (5.7 mg reduction for stem to leaf, 2.5 mg for leaf to stem) can be attributed primarily to internal bleeding. The 20 compressions were more effective at reducing leaf latex than six compressions (without abrasion and VEG acid) in the previous experiment when the 20 compressions were made from stem to leaf (*P* = 0.002, *t*-test), but not from leaf to stem (*P* = 0.29, *t*-test). Even with 20 compressions, substantial latex remained in the leaf, which likely explains why *T*. *zethus* larvae often make multiple girdles/furrows from the petiole towards the leaf tip. Each causes partial drainage of the remaining distal latex, thus reducing latex pressures in laticifers where the larva eventually feeds.

### Chemistry of VEG secretion

HPLC analysis documented the presence of formic acid (6.53 ± 0.83 M) and butyric acid (0.05 ± 0.01 M) in the VEG secretion from last instar *T*. *zethus*. Acetic acid, previously identified in the VEG secretion of other notodontids [37], was not detected, nor was propionic acid or isobutyric acid. The natural and reconstituted acid solutions produced similar pH readings (0.91 ± 0.05 for VEG secretion, 1.09 ± 0.02 for acid solution). However, these readings are outside the calibration range of the pH buffers used (pH 1.68 and 4.01) and thus should be interpreted with caution.

**Fig 5:**
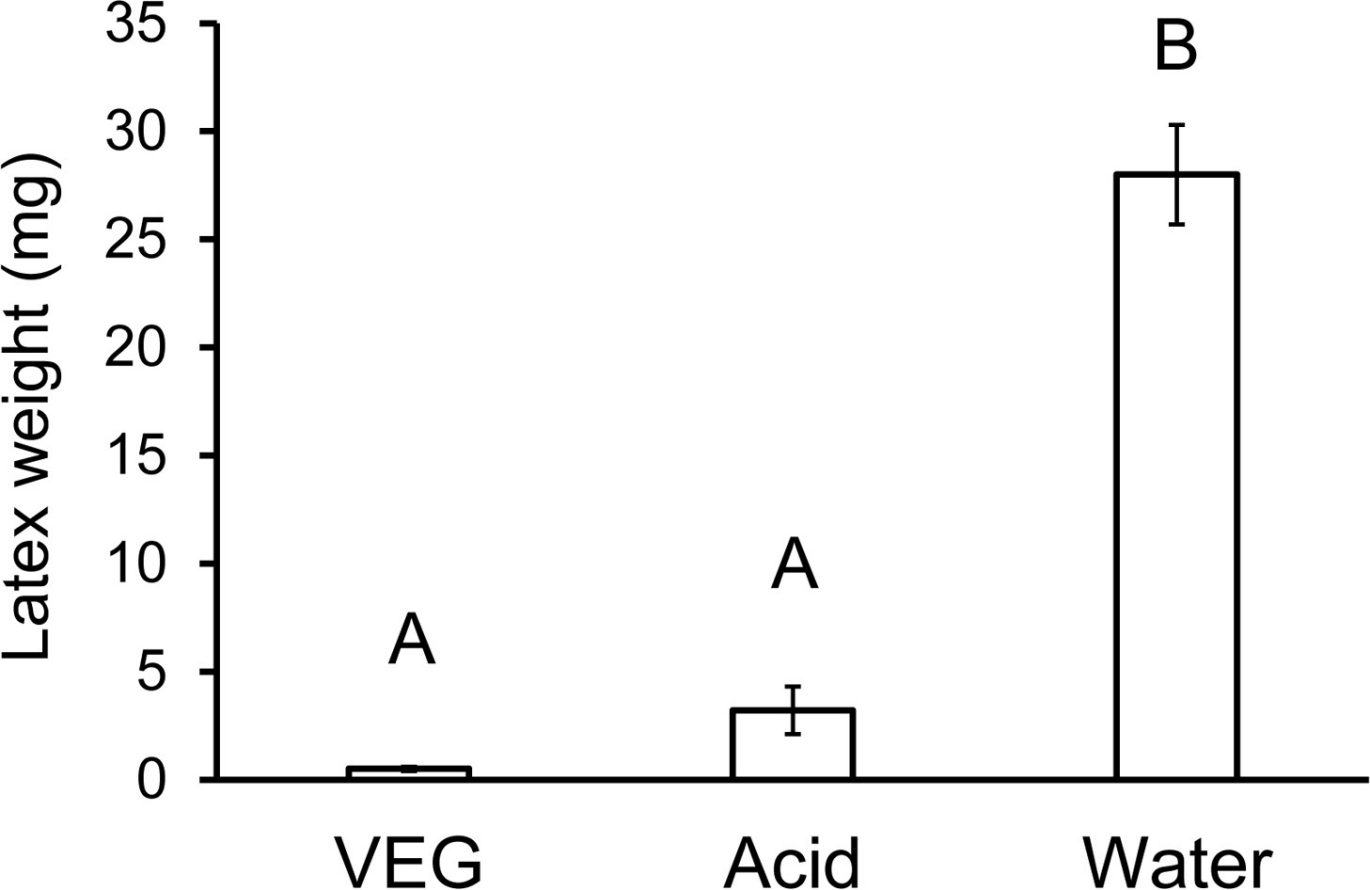
Effects of VEG secretion and acid constituents on latex outflow from poinsettia petioles. Petioles were severed 2.5 hours after being abraded and treated either with *T*. *zethus* VEG secretion, a reconstituted acid solution (6.53 M formic acid, 0.05 M butyric acid), or water. The VEG secretion and reconstituted acid solutions caused a similar reduction in wet weights of latex. Bars with different letters differ significantly at *P* < 0.05 using Games-Howell pairwise tests.

**Fig 6:**
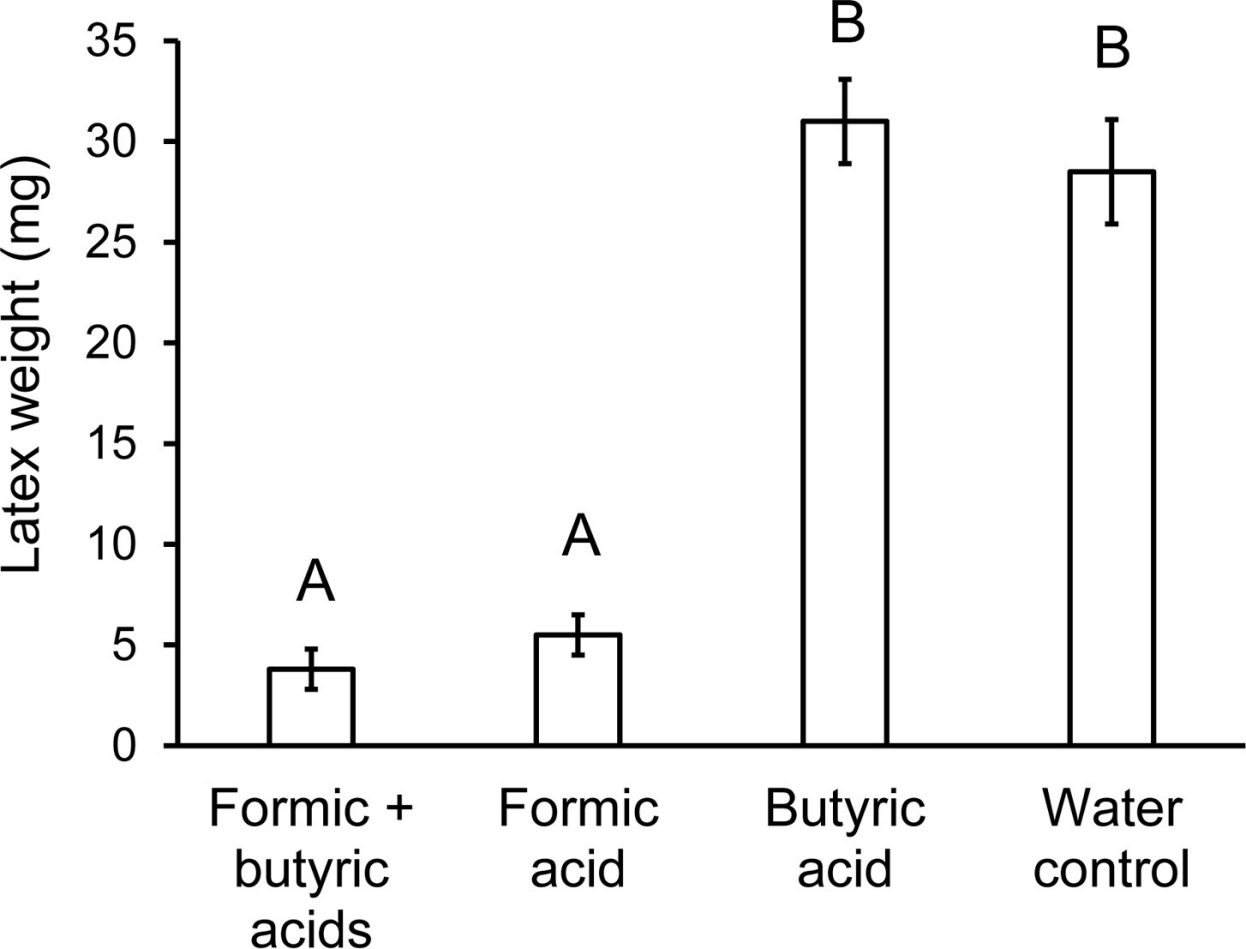
Effects of formic acid and butyric acid on latex outflow from poinsettia petioles. Petioles were abraded and treated either with 6.53 M formic acid + 0.05 M butyric acid, 6.53 M formic acid alone, 0.05 M butyric acid alone, or water. After 2.5 hours, the wet weights of latex emitted by severed petioles were measured. Formic acid alone caused a similar reduction in latex outflow as formic and butyric acid combined. Butyric acid by itself at low concentration did not decrease outflow. Bars with different letters differ significantly at *P* < 0.05 using Games-Howell pairwise tests.

When placed on poinsettia petioles, the reconstituted acid solution caused withering and reduced latex flow similar to *T*. *zethus* VEG secretion (Fig 5). The VEG secretion spread more readily over the dorsal petiole than the reconstituted acid solution, presumably because of minor lipophilic constituents noted in other notodontid VEG secretions [35, 37]. Formic acid alone decreased latex exudation as effectively as formic and butyric acid combined (Fig 6). The low concentration of butyric acid by itself had no detectable effect.

### Histology of *T*. *zethus* furrows

**Fig 7:**
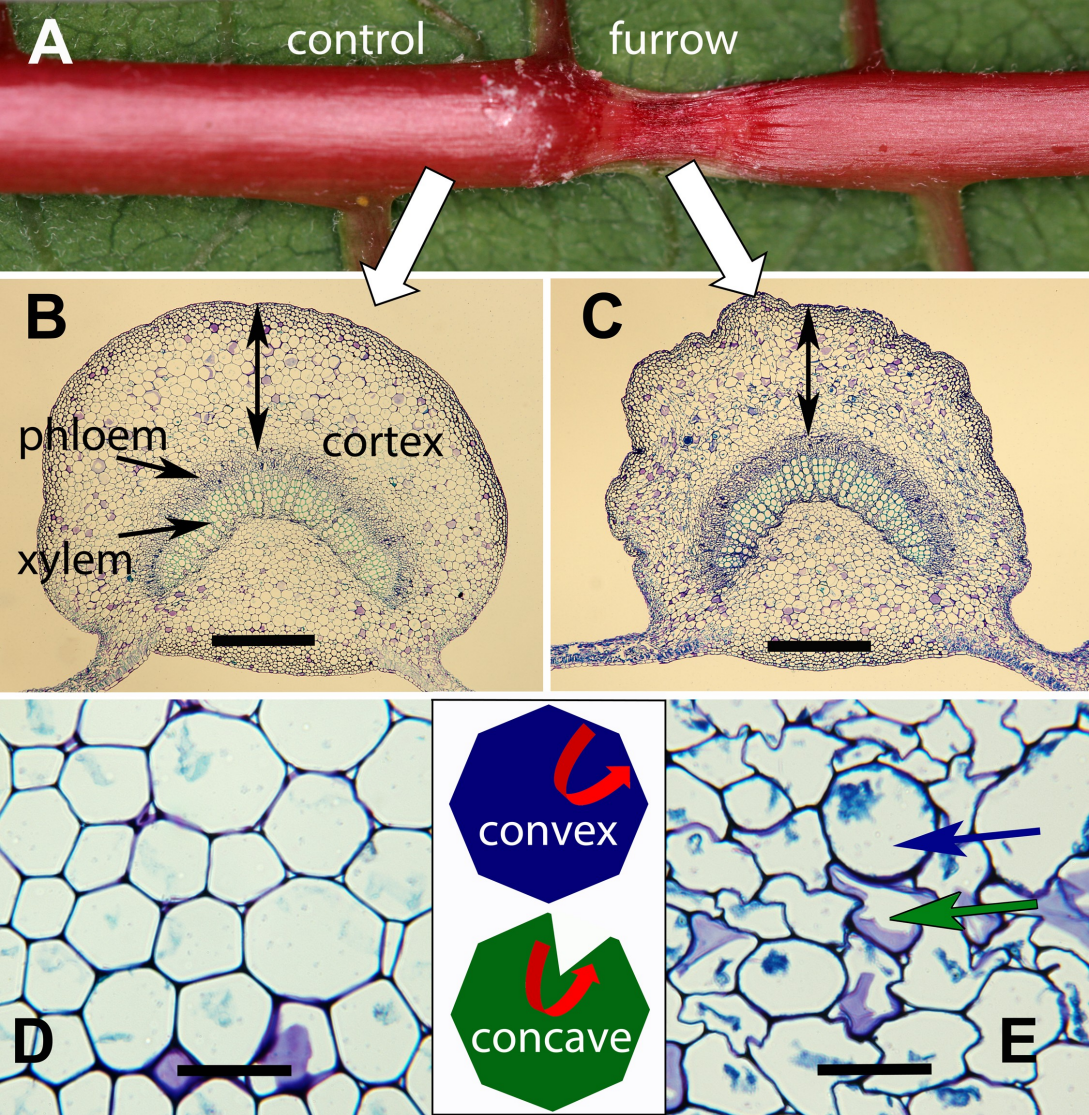
Effect of *T*. *zethus* furrows on cell morphology. (A) Furrow in a poinsettia midrib created by an intact control *T*. *zethus* larva. (B, C) Cross sections through the same poinsettia midrib outside the furrow (left) and in the center of the furrow (right). The black vertical lines represent transects from the phloem to the epidermis, which were used to count the number of cells with concave or convex shapes. Convex cells have round walls with all interior angles ≤180°; concave cells have at least one interior angle >180°. (D, E) Cortical cells outside (left) and within (right) cross sections of the same furrow viewed at higher magnification. An example of a convex cell (blue arrow) and a concave cell (green arrow) are marked in the bottom right image. The black scale bars equal 0.5 mm (B and C) or 0.05 mm (D and E).

**Fig 8:**
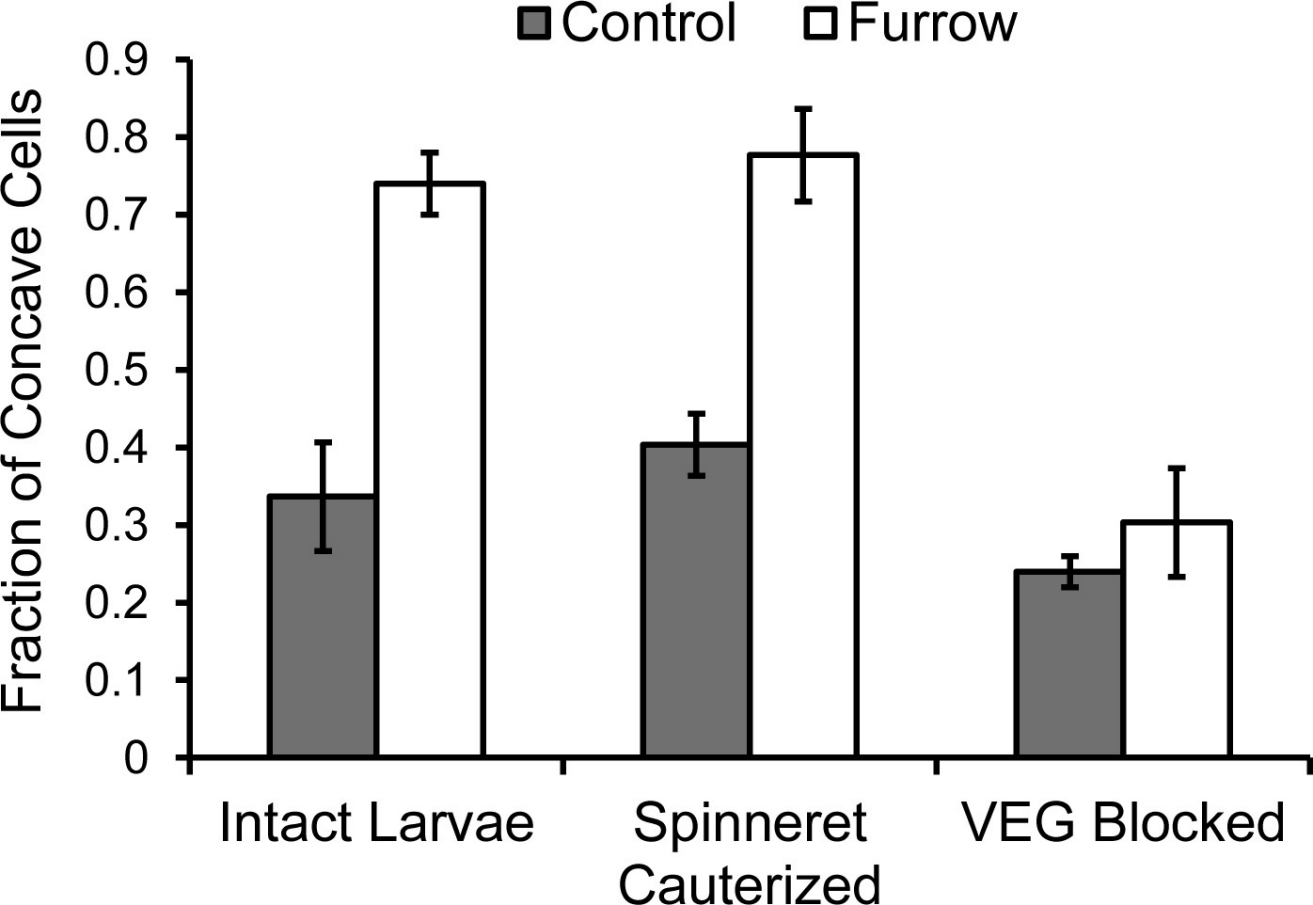
Distortion of poinsettia cell walls within and outside *T*. *zethus* furrows. The fraction of cells with a concave shape in midrib cross sections was measured along a transect from the phloem to the epidermis. Significantly more concave cells were present inside furrows than outside furrows for intact *T*. *zethus* larvae (*P* < 0.01, *t*-test) and larvae with cauterized spinnerets (*P* < 0.05), but not for larvae with a blocked VEG that were unable to release acid (*P* = 0.55).

Dehydrating and clearing furrows increased their width and depth (Fig 7A–7C, [44]), apparently by rehydrating and expanding cells. Nevertheless, larvae with blocked and functional VEGs created furrows that differed substantially in cell morphology (Figs 7 and 8). The intact controls and spinneret-cauterized larvae disrupted cell walls resulting in significantly more concave cells inside the furrow than outside the furrow (*P* < 0.05 paired *t*-tests). Interestingly, the cell walls remained intact, but were often grossly distorted especially for large interior cells in the cortex. In contrast with VEG-blocked larvae, cells within and outside “furrows” did not differ with most exhibiting a round shape (Fig 8).

### Related behaviors in other notodontid species

**Fig 9:**
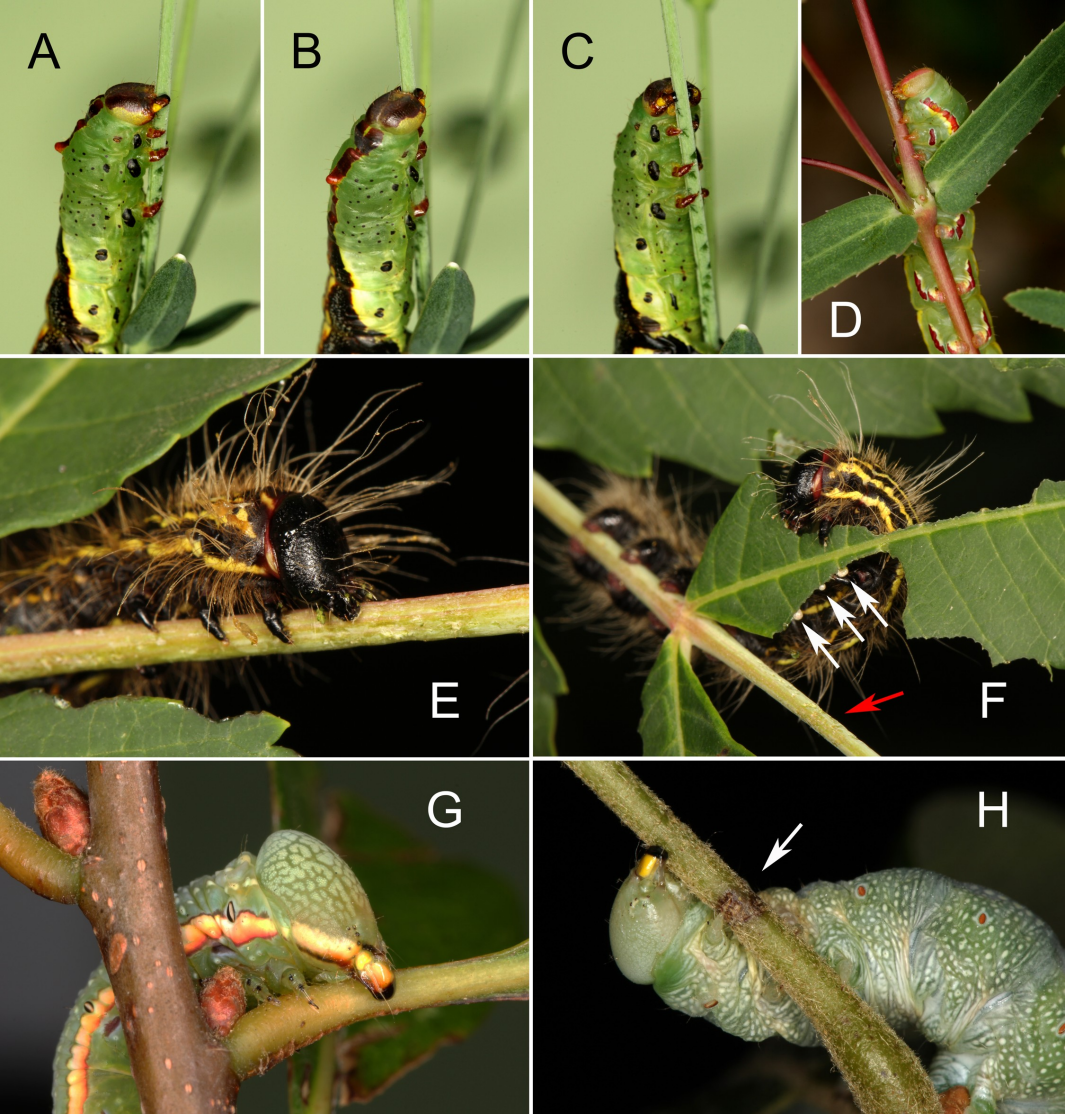
Stem and petiole compression by final instar notodontid caterpillars. (A-C) *Theroa zethus* larva walking along a *Euphorbia corollata* stem while compressing the stem from all angles. The dark green compressions are clearly visible. (D) *Praeschausia zapata* larva creating a girdle in a *Chamaesyce hyssopifolia* stem by compressing the stem repeatedly from all sides with its mandibles. (E) *Datana perspicua* larva compressing the rachis of a smooth sumac leaf (*Rhus glabra*). Hairs on the larva are glued together by exudate. (F) *D*. *perspicua* feeding on a smooth sumac leaf. Scars from previous rachis compressions (red arrow) and drops of white exudate (white arrows) are visible. (G) *Paraeschra georgica* larva compressing the petiole of a water oak leaf (*Quercus nigra*). (H) *Nadata gibbosa* larva compressing the petiole of a southern red oak leaf (*Q*. *falcata*). A brown scar from earlier compressions is indicated by the white arrow.

None of the six notodontid species were observed to scrape the plant surface and secrete VEG acid, although more extensive observations will be required to determine if these behaviors are truly absent, especially with *Praeschausia zapata*. However, all six species did use their mandibles for compression. *Praeschausia zapata*, like *T*. *zethus*, compressed the narrow petioles and stems of euphorb hosts (Fig 9A–9D, S5 Movie, [8]). Likewise, final instar larvae of *Datana perspicua* constricted the rachis of smooth sumac (*Rhus glabra*) (Fig 9E), presumably thereby disrupting the intercellular elongate ducts [45] that emit white exudate (Fig 9F). All four oak-feeding notodontids similarly compressed oak midribs or petioles. The final instar larvae of *Peridea angulosa*, for example, often constricted petioles of several adjacent water oak leaves, then initiated feeding on one of them (S6 Movie). The oak feeders often rotated around the petiole to compress the petiole from multiple directions (Fig 9G and 9H, S6–S8 Movies), just like *T*. *zethus* and *Praeschausia zapata*.

The four oak feeders also exhibited behaviors not observed with *T*. *zethus*. After consuming entire oak leaves except for the midribs, three species sometimes (*L*. *manteo and Paraeschra georgica*) or routinely (*Peridea angulosa*) severed the midrib or petiole with their mandibles. They then rubbed their labium over the stub (S9 Movie), thereby applying fluid, presumably saliva from the spinneret (as documented with notodontid girdling [50]). Leaf clipping has been described previously in *L*. *manteo*, *P*. *angulosa* [43] and other notodontids, plus eleven other families of caterpillars and sawflies [11, 51–56]. Fluid application to the midrib or petiole stub occurs in many species (DE Dussourd unpub. obs.). *Nadata gibbosa* larvae did not clip leaves, but instead chewed furrows in the midribs of both post oak and southern red oak leaves, then rubbed their labium over the furrow surface (S10 Movie). The midrib furrows only superficially resembled the acid furrows of *T*. *zethus*. *Nadata gibbosa* larvae created furrows by consuming a portion of the midrib, whereas *T*. *zethus* caterpillars made furrows with acid and compression. The function of the girdling, furrowing and leaf-clipping behaviors of notodontids on hardwoods remains unresolved, although in each case the larvae expose vascular tissues then appear to coat the cut surface with saliva ([11, 50] DE Dussourd unpub. obs.). Fluid application appears to be less substantial during petiole compression (S5–S8 Movies).

## Discussion

### Sabotage of latex defense

Whether an herbivore can feed on a particular plant species is determined not just by the array of defenses present in the plant, but also by the herbivore’s ability to tolerate, circumvent, or deactivate the defenses [11, 57]. Biochemical adaptations for metabolizing and excreting allelochemicals, for preventing uptake, or for sequestering them in safe repositories have been documented in numerous insect species [58, 59]. This study demonstrates instead the synergistic effects of insect behavior and glandular secretions on plant defenses. *Theroa zethus* larvae sabotage the latex canals of their euphorb hosts by repeatedly secreting VEG acid onto the stem, petiole, or midrib surface. Acid penetration through the waxy cuticle is facilitated by mandibular scraping of the plant surface. With their cutin and wax barriers to ion diffusion [60, 61], intact cuticles are nearly impermeable to hydrogen ions [62]. The apparent absence of laticifers in the poinsettia epidermis [25] enables larvae to abrade the cuticle without causing latex emission. Multiple rounds of scraping and acid application soften tissues allowing larvae to grip and compress the midrib or petiole (S3 Movie). Poinsettia laticifers are bounded by a primary cell wall usually only slightly thicker than the walls of adjacent parenchyma cells [25]. The combination of low pH and compression ruptures the laticifers internally, thus preventing latex flow to distal areas of the leaf where the larva initiates feeding. *Theroa zethus* larvae on poinsettia complete a furrow in 39 ± 10 minutes [8]. Even after 2.5 hours, VEG acid by itself did not reduce latex outflow from petioles (Fig 2), but the combination of VEG acid with abrasion and constriction decreased outflow by 97% in an hour (Fig 3).

Compression of acid-treated petioles blocked latex movement from the stem into the leaf, but was less effective at draining latex from the leaf blade (Figs 3 and 4) perhaps because the petiole has little internal space for receiving latex (Fig 7). If *T*. *zethus* larvae were instead to cut into the petiole with their mandibles, latex would drain onto the surface, which would more effectively reduce leaf latex pressures, but the larvae would directly contact exudate and experience its adhesive and poisonous properties. Late instar danaines and *Erinnyis* sphingids often encounter and ingest latex while constricting and chewing into the petiole or midrib of their laticiferous host plants [11, 63–65]. First instar *T*. *zethus* likewise cause large drops of latex to exude while cutting into *Chamaesyce maculata* midribs with their mandibles [8]. Beetles and katydids on Euphorbiaceae and Apocynaceae minimize latex contact by biting veins with powerful mandibles, then moving away from exuding latex. Both the beetles and katydids sever veins repeatedly; each successive cut is made distal to earlier cuts, thus repeatedly draining latex from their eventual feeding site [64, 66].

On euphorbs with narrow stems, *T*. *zethus* larvae likewise compress stems in an apical direction, rotating around the stem to constrict it from multiple angles (Fig 9A–9C, [8]). Compressions made from stem to leaf more effectively drained leaf latex than compressions from leaf to stem (Fig 4). Stem-to-leaf compressions repeatedly rupture laticifers extending into the leaf, thereby draining more and more latex from the leaf where the larva eventually feeds. With leaf-to-stem compressions, the first compressions rupture the laticifers, thus isolating laticifer branches in the leaf from additional compressions made closer to the stem. Compressions caused minimal surface exudation suggesting that the reduction in leaf latex can be attributed specifically to internal bleeding. The occasional spontaneous emission of latex onto the petiole surface (seen in the S5 Movie of *Praeschausia*) provides evidence that the internal latex is not clotted, but remains fluid during compression. Whether *T*. *zethus* acid or saliva cause clotting of internal or exuded latex is not known. Draining latex from leaves is particularly challenging with poinsettia due to the thick petioles and large leaves containing sizable reservoirs of latex [67]. *T*. *zethus* larvae reduced latex pressures in poinsettia leaves by creating multiple girdles and furrows, each produced distal to a previous one. The larvae sometimes also constricted and cut individual side veins, which combined with girdles and furrows reduced exudation where they fed [8].

### Distortion of cell walls within *T*. *zethus* furrows

Plant cells exposed to highly acidic conditions suffer membrane damage and loss of turgidity [62], which could cause the observed withering in *T*. *zethus* girdles and furrows [8]. The acid growth hypothesis suggests an additional possible mechanism for how *T*. *zethus* larvae disrupt laticifers [8]. Growing plants expand cells by loosening their walls through the secretion of protons into the apoplast. Low pH activates proteins (expansins) and enzymes that allow the cell wall to be stretched [62, 68]. Acidic conditions also weaken cell walls by degrading hemicellulose [69]. Loosening of cell walls can occur rapidly; coleoptiles and hypocotyls exposed to pH 3.0–4.9 buffer solutions exhibited elongation growth almost immediately [62, 70]. Interestingly, with cotyledons abraded to enhance proton uptake, the greatest increase in cell wall extensibility occurred at the lowest pH tested (pH 2) [71].

*Theroa zethus* VEG secretion may similarly cause rapid acid-mediated weakening of cortical and laticifer cell walls. Cross sections through midrib furrows documented that internal cells often exhibited deformed walls (Fig 7). These deformities were not present in midribs treated by larvae with blocked VEGs indicating that the distortions can be attributed specifically to VEG acid or to the combination of VEG acid and compression. Laticifers store latex under considerable pressure [29, 30]; latex surges from the laticifers when they are damaged. Wounds are not always required for rupture. Lettuce laticifers sometimes spontaneously burst under high-growth conditions leading to a disorder known as tipburn where latex spilling out of laticifers causes localized necrosis in the leaves [72, 73]. Acid from VEG secretion might similarly weaken laticifer walls making them vulnerable to rupture. Given that VEG acid by itself reduces latex emission (Fig 3), laticifer impairment is clearly not due exclusively to mandibular constriction. The low pH of VEG acid does not appear to kill the elongate multinucleate laticifer cells passing through girdles and furrows, but only to cause localized damage with limited drainage of latex from surrounding areas. The laticifer cells remain functional; they still emit latex when either the adjacent stem or leaf is injured.

### Chemistry of VEG secretion

The VEG secretion of *T*. *zethus* contains primarily formic acid with small amounts of butyric acid, a previously unreported constituent of notodontid VEG secretions. A reconstituted solution of formic and butyric acid reduced latex outflow with similar effectiveness as VEG secretion (Fig 5), documenting that the acids cause laticifer disruption. Formic acid has been previously noted in the VEG secretions of other notodontid species at concentrations comparable to levels in *T*. *zethus* (6.53M = 30%). The VEG secretion of *Cerura* contains 37.5% formic acid [42], whereas the VEG of *Lochmaeus manteo* has 20–37% [41]. Notodontid VEG secretions have been reported to deter a variety of potential predators, including birds, lizards, toads, spiders and ants, although these claims appear to be supported exclusively by unpublished data [41, 74]. Nevertheless the use of the VEG for predator deterrence appears to be widespread in the Notodontidae [75], but only *T*. *zethus* is known to use its VEG for deactivating plant defenses.

### Evolution of *T*. *zethus* host choice

The overwhelming majority of the >3,500 described species of notodontids worldwide are thought to feed on woody trees and shrubs [7, 20, 22, 76–79], which largely lack laticifers [47, 48]. *Theroa zethus* and *Praeschausia zapata* are highly unusual in feeding on euphorbs, which differ in taxonomy, defenses, growth habit, and life history from the typical host plants of notodontids. These two species are members of the derived subfamily Heterocampinae within the Notodontidae [80]. How did the host shift occur in the *Theroa* and *Praeschausia* lineage(s) from the presumed woody ancestral food plants to herbaceous euphorbs protected by latex canals?

The sophisticated behaviors that notodontid larvae employ for modifying hosts may have facilitated this shift. Girdling, furrowing, and leaf-clipping behaviors occur in multiple notodontid subfamilies [11, 43, 53]. In all three behaviors, caterpillars on hardwood trees use their mandibles to cut directly into plant tissues. Similar cuts in plants with laticifers would expose a larva to direct contact with toxic, sticky exudate. Mandibular constriction by *T*. *zethus*, in contrast, elicits minimal latex release. On plants with narrow stems or petioles, compressions suffice to block latex flow and to reduce distal latex exudation (Fig 4, [8]). Notodontid larvae from diverse groups on hardwood trees similarly constrict the petioles of leaves (Fig 9). Petiole constriction on hardwoods, described here apparently for the first time, presumably serves to trap photosynthates in the leaf by disrupting vascular transport and/or to eliminate the movement of signaling molecules and defensive compounds. The capability of diverse notodontids to constrict petioles and thereby disrupt vascular tissues and any associated laticifers or ducts may have facilitated the host shift in the *Theroa* lineage onto latex-bearing plants. In this case, petiole constriction can be viewed as a pre-adaptation that now serves a new function on laticiferous plants: disarming the latex defense.

Behavioral circumvention or deactivation of host defenses has been documented in many lepidopteran lineages besides notodontids [11, 57]. Notable examples include taxa that sever veins or cut trenches [10, 65], mow trichomes [63, 81–84], or tie leaves and hide in the leaf shelter by day, thus preventing photoactivation of plant toxins [85]. Such behavioral adaptations not only allow larvae to consume otherwise unpalatable hosts, they might also facilitate host range expansion. General-purpose behaviors like petiole compression, vein cutting, trenching, or leaf tying could be utilized on diverse hosts and might allow an egg laid on an atypical host to complete development. Most vein cutting and trenching insects are dietary specialists [65], like most insect herbivores [1], but a few species feed on distantly related hosts that share the presence of latex canals. In the danaines, for example, the ability to cut veins and trench may have allowed some species to expand their host range beyond milkweeds to include latex-producing plants in the Moraceae and Caricaceae [65]. We propose that behavioral adaptations such as petiole compression or trenching could often be critical elements in ecological fitting, where an herbivore is not perfectly adapted to a novel host, but able to survive, leading to the eventual inclusion of the new host within an expanded host range [86, 87].

## Supporting information

S1 Data Raw data for each experiment. (XLSX)

S1 Movie Final instar *T*. *zethus* larva abrading the petiole of poinsettia, *Euphorbia pulcherrima* and applying VEG acid. The larva repeatedly alternated between abrasion and VEG application. This short clip shows the larva applying VEG acid, then scratching the petiole surface with its mandibles before applying acid again. Film speed has been increased 2x. The behaviors of *T*. *zethus* larvae are similar on poinsettia and on natural hostplants (S2 and S4 Movies).
(MP4)

S2 Movie Final instar *T*. *zethus* larva abrading the stem of a potted *Chamaesyce hyssopifolia* stem. Scratch marks from mandibular teeth are clearly visible. After abrading the stem surface, the larva pressed its VEG opening against the surface to release acid onto the scratches.
(MP4)

S3 Movie Final instar *T*. *zethus* larva compressing a poinsettia petiole with its mandibles. Compression ruptures latex canals, thus preventing latex flow to the leaf and draining some latex from the leaf.
(MP4)

S4 Movie Final instar *T*. *zethus* larva on *Euphorbia cyathophora* compressing the petiole. Film speed has been increased 2x.
(MP4)

S5 Movie Final instar *Praeschausia zapata* larva compressing a *Chamaesyce nutans* stem with its mandibles. Application of VEG acid was not apparent. Although the compressions sometimes caused latex emission, they reduced exudation during subsequent larval feeding on the leaf. Film speed has been increased 2x.
(MP4)

S6 Movie Final instar *Peridea angulosa* larva compressing the petiole of a water oak leaf, *Quercus nigra*. (MP4)

S7 Movie Final instar *Lochmaeus manteo* larva compressing the petiole of a water oak leaf, *Quercus nigra*. Film speed has been increased 3x.
(MP4)

S8 Movie Final instar *Nadata gibbosa* compressing the petiole of a southern red oak leaf, *Quercus falcata*. (MP4)

S9 Movie Final instar *Peridea angulosa* larva severing the petiole of a water oak leaf (*Quercus nigra)* after consuming the entire leaf blade except for the midrib. After clipping the petiole, the larva rubbed its labium over the stub apparently applying saliva. Previously before starting to feed, the larva compressed all three petioles visible in the video, thus creating the discolored brown sections. The larva subsequently severed the petiole at the same location that it had previously compressed.
(MP4)

S10 Movie Final instar *Nadata gibbosa* chewing a furrow in the midrib of a post oak leaf (*Quercus stellata*). While cutting the furrow, the larva periodically paused to rub its labium over the cut surface apparently applying saliva. Afterwards, the larva resumed feeding on the same side of the leaf as previously. Film speed has been increased 4x.
(MP4)
